# Low ambient temperature and sudden temperature drop increases the incidence of acute aortic dissection: a retrospective analysis from the northeast region of China

**DOI:** 10.3389/fpubh.2026.1848286

**Published:** 2026-05-29

**Authors:** Zhenyu Liao, Zhaorui Liu, Yue Ding, Ying Liu, Tao Song, Yike Wang, Ning Zhang, Chao Liu, Zhaoxin Fan, Xin Zhang, Haiyu Zhang, Song Zhang

**Affiliations:** 1 Department of Cardiology, The First Affiliated Hospital, Harbin Medical University, Harbin, China; 2 Department of Anesthesiology, Heilongjiang Provincial Hospital, Harbin, Heilongjiang, China; 3 Department of Neurosurgery, The First Affiliated Hospital, Harbin Medical University, Harbin, China; 4 Heilongjiang Provincial Center for Disease Control and Prevention, Harbin, China; 5 Key Laboratory of Cardiovascular Disease Acousto-Optic Electromagnetic Diagnosis and Treatment in Heilongjiang Province, The First Affiliated Hospital of Harbin Medical University, Harbin, China; 6 NHC Key Laboratory of Cell Transplantation, The First Affiliated hospital of Harbin Medical University, Harbin, Heilongjiang, China; 7 Key Laboratory of Cardiac Diseases and Heart Failure, Harbin Medical University, Harbin, China; 8 National Key Laboratory of Frigid Zone Cardiovascular Diseases, Harbin Medical University, Harbin, Heilongjiang, China

**Keywords:** acute aortic dissection, ambient temperature, daily mean temperature, risk factors, temperature change

## Abstract

**Background:**

Acute aortic dissection (AAD) is a cardiovascular catastrophe with substantial early mortality. Although ambient cold has been implicated as a trigger, existing evidence derives predominantly from temperate and subtropical settings. Populations enduring protracted severe winters–a hallmark of cold temperate zones–remain underrepresented, leaving the cardiovascular hazard of sustained extreme cold poorly defined.

**Methods:**

In this retrospective time-stratified case-crossover study, we analyzed 1 384 patients with AAD admitted to a tertiary cardiovascular center in Harbin, China–a city in the cold temperate zone of Northeast Asia with a harsh continental monsoon climate. Meteorological data (2014–2023) were interrogated using distributed lag non-linear models combined with conditional logistic regression to quantify exposure lag-response relationships between daily mean temperature and temperature change between neighboring days (TCN) with the AAD onset.

**Results:**

A near-linear increase in AAD risk was observed with decreasing daily mean temperature, with minimum morbidity at 25.9 °C. Extreme cold (−23.4 °C, 1st percentile) raised the risk significantly within 3 days. A pronounced risk elevation followed temperature drops between consecutive days–a decline of 9.2 °C was associated with a RR of 9.57 (95% CI 1.21–75.76). In contrast, temperature rises conferred protection. These effects were accentuated in winter and during the period of centralized indoor heating.

**Conclusion:**

In a cold temperate region marked by severe winters, both low ambient temperature and abrupt cooling act as potent environmental triggers for AAD. These findings call for enhanced clinical awareness and tailored preventive measures during cold exposure in such climates.

## 1 Introduction

Acute aortic dissection (AAD) is a highly lethal aortic disease, with population-based studies reporting an annual incidence of approximately 3 to 20 cases per 100,000 individuals worldwide (1, 2). A prospective cohort study from the China Kadoorie Biobank (*n* = 512,724) reported an overall incidence of 2.35 per 100,000 person-years (3.97 in men and 1.25 in women), identifying hypertension and male sex as independent risk factors, while body mass index and diabetes showed no significant association (3). Despite advances in medical and surgical management, early mortality remains exceedingly high, especially within the initial hours post-onset, and in-hospital mortality can still exceed 20% (4). Therefore, identification of modifiable triggering factors is crucial, as targeted prevention strategies hold significant potential for reducing the incidence of this catastrophic condition (5).

Among environmental determinants, ambient temperature has drawn interest for its established link to cardiovascular events such as myocardial infarction (6, 7) and stroke (8). Epidemiological evidence indicates that AAD incidence varies seasonally, occurring more frequently in colder months (9, 10). Recent multi-center and nationwide studies also consistently report that low ambient temperatures and temperature variability elevate the risk of AAD onset (11–13). However, a critical limitation persists: the vast majority of this evidence originates from temperate, subtropical, or tropical regions (10). The environmental stressors, population acclimatization, and behavioral patterns in these regions differ profoundly from those in cold temperate zones characterized by prolonged, severe winters (14). Consequently, the exposure-response relationships, lag patterns, and potentially even the underlying physiological triggers for AAD identified in milder climates may not be directly applicable to populations experiencing sustained extreme cold and sharp indoor-outdoor temperature transitions.

Despite the clear biological plausibility that extreme cold is a potent cardiovascular stressor, data quantifying its association with AAD from authentic cold temperate zones remain strikingly scarce. Large-scale registries, such as the Registry of Aortic Dissection in China (Sino-RAD), have included limited representation from these high-latitude regions (15, 16). This gap leaves clinicians and public health strategists without region-specific evidence regarding how the unique climatic extremes—combining sustained low temperatures, dramatic temperature drops, and artificial heating-induced thermal contrasts—influence the epidemiology of AAD.

Harbin, the capital of Heilongjiang Province in northeastern China, is a major city in the cold temperate zone. It experiences a harsh continental monsoon climate, with winter temperatures often remaining below −20 °C for extended periods (17). A distinctive feature is its half-year-long centralized heating system, which creates substantial indoor-outdoor temperature gradients. This setting offers a unique opportunity to investigate the impact of severe, sustained cold exposure on AAD in a real-world, high-risk population.

This study examined the association between short-term exposure to low ambient temperature, temperature change between neighboring days (TCN), and AAD onset in a population from a cold temperate zone in the northeast of China. Using a time-stratified case-crossover design, we evaluated both immediate and lagged effects of cold exposure on AAD risk. The findings aim to address an important climatic and geographic gap in the literature and to inform region-specific preventive strategies for this life-threatening condition.

## 2 Methods

### 2.1 Study design

In this study, we applied an individual-level, time-stratified case-crossover design to explore the association between short-term exposure to low ambient temperature and TCN, and the onset of AAD in a cold region in northeastern China. Because each case serves as its own control, this design avoids bias from control selection and inherently controls for time-invariant confounders (such as age, sex, chronic diseases, and socioeconomic status), as well as day-of-week effects and long-term trends (18, 19). For each participant, the date of AAD occurrence was defined as the case day, and control days were selected from dates within the same year and month that fell on the same day of the week. For example, if a case occurred on October 11, 2016 (a Tuesday), the corresponding control days were October 4, 18, and 25, 2016.

### 2.2 Study population

We enrolled patients who diagnosed with acute aortic syndrome in the First Affiliated Hospital of Harbin Medical University (Harbin, China) from 2014 to 2023. The First Affiliated Hospital of Harbin Medical University is a Tertiary A hospital and serves as the largest medical center for cardiovascular diseases in Heilongjiang Province. The clinical staging of aortic dissection follows the standards established by the Society for Vascular Surgery (SVS) and the Society of Thoracic Surgeons (STS) (20), which define the acute phase as 1–14 days, the subacute phase as 15–90 days, and the chronic phase as >90 days. A retrospective review of electronic medical records was conducted to identify patients discharged with a diagnosis of acute aortic syndrome. Diagnostic modalities included computed tomography angiography and transthoracic echocardiography performed at admission. After excluding conditions with similar presentations, such as aortic aneurysm and penetrating aortic ulcer, and records lacking information, the patients meeting the diagnostic criteria for acute aortic dissection (AAD) were included in the final analysis. This study was approved by the Ethics Committee of the First Affiliated Hospital of Harbin Medical University (Approval No. 202639), with waiver of informed consent due to the de-identified nature of the data.

Heilongjiang Province is situated in the northeast of China, geographically located in a frigid zone. It borders the Far Eastern Siberian region of Russia to the north, and it intersects with the primeval forest of the Greater Khingan Mountains on the west. Harbin city, the provincial capital of Heilongjiang, features a typical continental monsoon climate characterized by long and cold winters, with daily average temperatures remaining below −20 °C for over 1 month (Supplementary Figure 1).

### 2.3 Data collection and exposure definition

Meteorological data for the study region between May 2014 to September 2023 were obtained from the Harbin Meteorological Bureau and six county-level meteorological stations distributed around Harbin City. The collected data included daily maximum, minimum, and average temperatures. The daily mean temperature was defined as the average of hourly temperature readings over a 24 h period (21). In previous epidemiological studies, daily mean temperature has been more commonly used to investigate the risk of cardiovascular diseases (22, 23). Furthermore, TCN is defined as the difference between the daily mean temperature of the day minus the daily mean temperature of the previous day. Previous studies had indicated that the temperature change between neighboring days (TCN) is associated with an increased risk of AAD (9, 10). Therefore, TCN was also calculated in this study. Here, a positive TCN value indicates that the current day's temperature is higher than that of the previous day, whereas a negative TCN value indicates that the current day's temperature is lower than that of the previous day.

Harbin employs a centralized heating system, which operates continuously for 6 months each year from October to March. During this period, the temperature difference between indoors and outdoors can reach as high as 40 °C (averaging −20 °C outdoors to 20 °C indoors). Here, we divided the period into central heating and non-central heating phases, as well as into four seasons (spring, summer, autumn, and winter) to compare the number of AAD cases across different time intervals.

## 3 Statistical analysis

Based on the outcome variable and the case-crossover design, a conditional logistic regression model was applied to examine the association of daily mean temperature and TCN with the incidence of AAD. To explore the potential non-linear and lagged effects of temperature and TCN on AAD incidence, we performed a distributed lag non-linear model (DLNM) to construct cross-basis functions for temperature and TCN, which were incorporated into the conditional logistic model (24, 25). In the cross-basis function for temperature, the exposure-response relationships were modeled using a B-spline function with three knots placed at the 10th, 75th, and 90th percentiles of the temperature distribution. Meanwhile, a natural cubic spline of 3 degrees of freedom (df) was applied for TCN (10, 21, 26). We adopted a maximum lag of 15 days to explore the lagged effect, and the natural cubic spline function was used with two knots placed at equal intervals on the log scale.

Subsequently, we plotted the exposure-response relationship curve across the temperature range from the 1st to the 99th percentile to decrease statistical uncertainty caused by the quite limited data at extreme exposures. We identified the minimum morbidity temperature (MMT) associated with the minimum incidence risk of AAD on the exposure-response curve, and calculated cumulative relative risks (RRs) and 95% confidence intervals (CIs) at different temperature. In the analysis of extreme cold and extreme heat effects, the temperatures were set at the 1st and 99th percentiles, respectively, with the MMT used as the reference. For the analysis of TCN, a value of 0 °C was directly used as the reference to calculate RRs and 95% CIs.

We further collected the daily average concentrations of ozone (O3) and fine particulate matter (PM2.5) from 2014 to 2023, incorporating them into the model sequentially and then simultaneously to adjust for potential confounding factors in sensitivity analyses. Stratified analyses were conducted based on age (< 60 years or ≥60 years) and sex (male and female).

All statistical tests were two-sided, and a value of *P* < 0.05 was considered statistically significant. We conducted statistical analyses in the R software (version 4.5.1, R Foundation for Statistical Computing, Vienna, Austria).

## 4 Results

### 4.1 Study population and baseline characteristics

**Figure 1:**
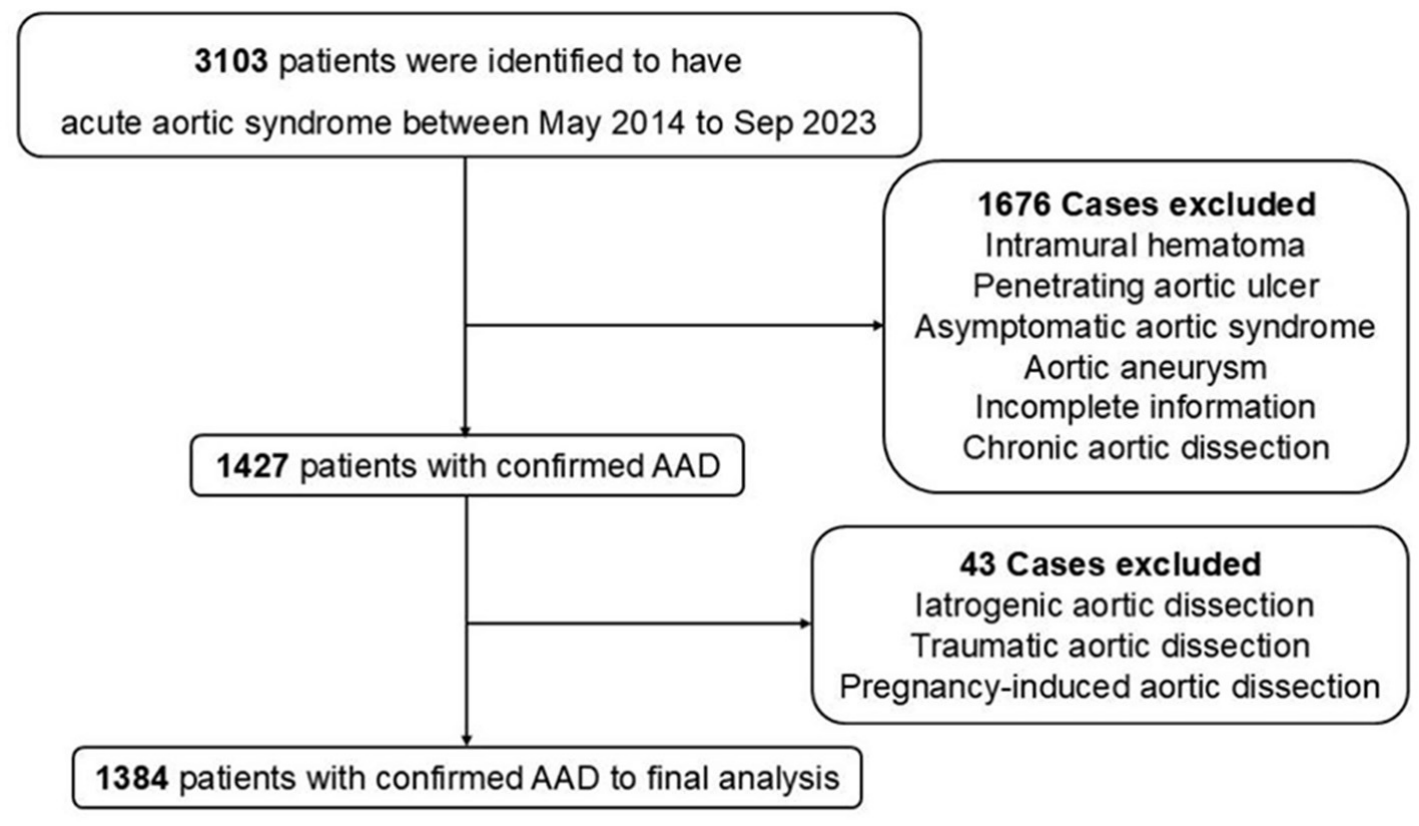
Patient selection flow diagram. The flow diagram shows the inclusion and exclusion and 1,384 AAD patients were included in the final study. AAD indicates acute aortic dissection.

From May 2014 to September 2023, a total of 3,103 patients were diagnosed with acute aortic syndrome at the First Affiliated Hospital of Harbin Medical University. After excluding 1,676 patients with intramural hematoma, penetrating aortic ulcer, asymptomatic aortic syndrome, aortic aneurysm, chronic aortic dissection and incomplete information and 43 patients with traumatic, iatrogenic, or pregnancy-induced dissections, we included 1,384 AAD patients in the final analysis (Figure 1). The majority of these patients were male and 65.3% were aged < 60 years. Most patients did not have coexisting hypertension or diabetes. The baseline information of all patients was shown in Table 1. The RRs for lower and higher temperature was estimated relative to the reference temperature of 25.9 °C. In this table, RRs are based on 15 days of temperature exposure (Supplementary Table 1). The temperature data corresponding to all observational dates included in the analysis are presented in the form of a histogram (Supplementary Figure 2).

**Table 1:** Baseline characteristics of study population.

Characteristics aged	Number	Proportion (%)
Sex		
Male	974	70.4
Female	410	29.6
Age, years		
< 60	904	65.3
≥60	480	34.7
Hypertension		
Yes	602	43.5
No	782	56.5
Type 2 diabetes		
Yes	45	3.3
No	1,339	96.7
All	1,384	100

**Figure 2:**
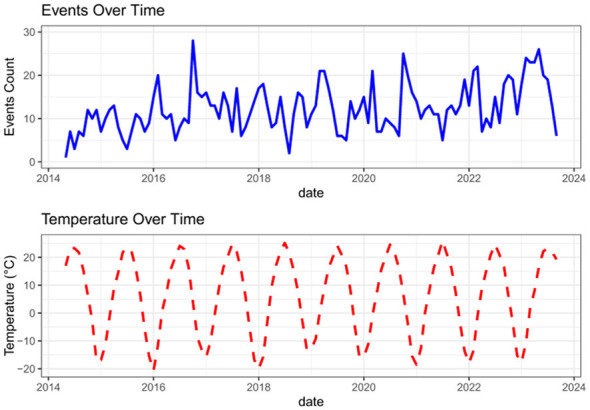
Time series distributions for daily mean temperature and monthly onset cases of acute aortic dissection during 2014–2023.

As shown in Figure 2, it illustrates the daily average temperature fluctuations and the number of AAD events in the region of our medical center from 2014 to 2023. It can be observed that the number of AAD events corresponds to the trend in daily average temperature. A higher number of AAD cases is noted during the colder months (Jan to Mar, Oct to Dec), whereas a lower number is observed in the warmer months (Apr to Sep) (Supplementary Figures 3A, C). In addition, the number of AAD cases during the heating period is also higher than that during the non-heating period (Supplementary Figure 3B).

### 4.2 Daily mean temperature and onset of AAD

**Figure 3:**
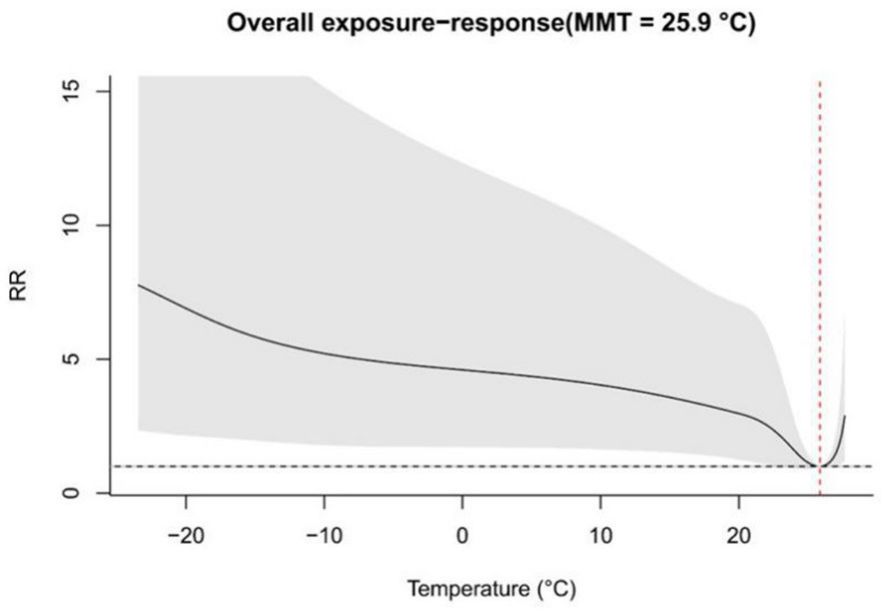
Exposure-response curve of daily temperature and risks of acute aortic dissection onset. Exposure-response curve of daily temperature and risks of acute aortic dissection onset cumulated over lag 0–15 day. Risk ratios and 95% confidence intervals were calculated based on a referent temperature of 25.9 °C. The black solid line is mean risk estimates and the gray areas are their 95% confidence intervals. The red vertical dotted line is 25.9 °C and denotes the referent temperature that has the statistically lowest risk.

**Figure 4:**
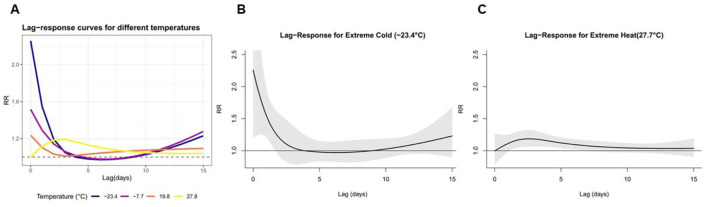
Lag-response curves for different temperatures. **(A)** The lag-response curve of different temperatures on the incidence of AAD for 1st percentile, 25th percentile, 75th percentile and 99th percentile. **(B)** The lag effect of daily mean temperature on the incidence of AAD at the extremely cold temperature (−23.4 °C, 1st percentile). The lag effect of daily mean temperature on the incidence of AAD at the extremely heat temperature (27.7 °C, 99th percentile). **(C)** The lag effect of daily mean temperature on the incidence of AAD at the extremely heat temperature (27.7 °C, 99th percentile).

The exposure-response curve showed that the lowest risk of AAD occurred at 25.9 °C, which was therefore defined as the reference temperature. The curve illustrates an almost linear exposure-response relationship between the lower daily mean temperatures with an increased risk of AAD, which indicates the risk was rising along with temperatures decreased. In contrast, no significant association was observed between higher temperatures and the risk of AAD (Figure 3). The contour and 3-dimensional maps comprehensively show the bidimensional expose-hysteresis response relationship between the incidence of AAD and daily average temperature (Supplementary Figures 4A, B). The extremely cold temperature (−23.4 °C, 1st percentile) significantly increased the risk of AAD within 3 days (2.252; 95% CI, 1.205–4.208 for lag 0; 1.545; 95% CI, 1.21–1.974 for lag 1; 1.194; 95% CI, 0.907–1.573 for lag 2), and the extremely hot temperature (27.7 °C, 97.5th percentile) had no significant effect on the onset risk of AAD within 15 days (lag 0 to lag 15) (Figure 4 and Supplementary Table 2).

### 4.3 TCN and onset risk of AAD

**Figure 5:**
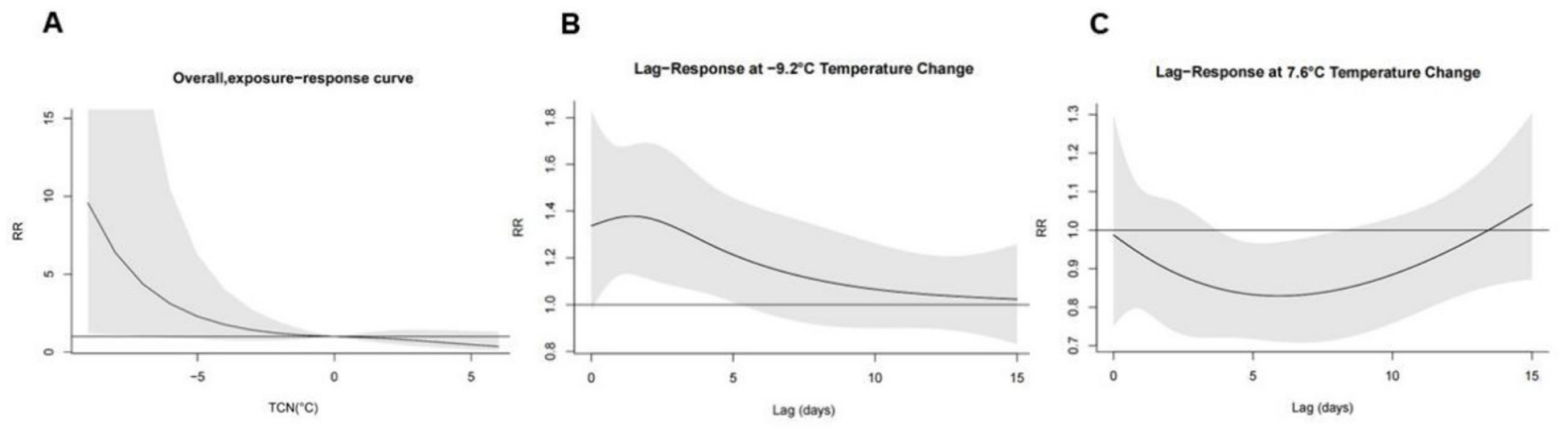
Lag-response curves for the risks of acute aortic dissection at different TCN. **(A)** Overall exposure-response curve for the risks of AAD at different TCN; **(B, C)** Lag-response curves for the risks of AAD at different TCN. The risks were presented as relative risk and 95% confidence intervals of acute aortic dissection at extremely negative temperature change between neighboring days [**(B)** −9.2 °C, 1st percentile] and extremely positive temperature change between neighboring days [**(C)** 7.6 °C, 99th percentile] compared to the referent temperature change between neighboring days (0 °C) on different lag days. The black solid lines are mean risk estimates and the gray areas are their 95% confidence intervals.

An association between TCN and the incidence of AAD was also observed. An inverse exposure–response relationship between TCN and AAD risk was evident over lag days 0–15 (Figure 5A). Negative TCN values, representing a temperature drop from the previous day, were associated with an increased risk of AAD, whereas positive TCN values, indicating a temperature increase, corresponded to a lower risk. For instance, compared to no temperature change, the RR of AAD for an extremely negative TCN (−9.2 °C) was 9.566 (95% CI: 1.208, 75.762), while that for an extremely positive TCN (7.6 °C) was 0.356 (95% CI: 0.097, 1.303) (Supplementary Table 3). The associations of extreme TCN with AAD were present on the concurrent day (lag 0) and persisted for up to 15 days (Figures 5B, C).

### 4.4 Stratified and sensitivity analysis

Stratified analysis showed that the point estimate of the RR associated with extreme temperature was tended to higher in the younger age group (< 60 years) compared with those aged ≥60 years, particularly under extreme low temperature conditions, although the difference did not reach statistical significance. In addition, female AAD patients were more susceptible to extreme temperatures than males, a pattern that was also observed for extreme low temperature conditions (Supplementary Table 4). In contrast, for extremely negative TCN, the risk of AAD appeared greater in patients aged ≥60 years and in females (Supplementary Table 5).

Results from sensitivity analyses were in general comparable to our main analyses. Estimates for lag-response with extreme temperature and TCN were similar with our main results after additionally controlling for PM2.5 and O3 (Supplementary Figures 5A–D).

## 5 Discussion

In this single-center, retrospective, time-stratified case-crossover study conducted in Harbin—a representative city in the cold temperate zone of Northeast China—we investigated the association between ambient temperature, abrupt temperature changes between neighboring days (TCN), and the incidence of AAD. Our findings demonstrate that in a region characterized by prolonged severe winters and significant indoor-outdoor temperature gradients, the risk of AAD exhibits an almost linear negative correlation with daily mean temperature, with the minimum morbidity temperature (MMT) identified at 25.9 °C, while the highest rate was found at −23.4 °C and with a 3-day lag effect. Furthermore, compared to the period before onset, a sharp temperature drop (−9.2 °C) was significantly associated with the occurrence of AAD, and the lag effect could persist for up to 15 days. These results not only corroborate prior nationwide observations but also extend the current understanding of temperature-related AAD risk to understudied climatic extremes.

Earlier epidemiological studies have indicated that the incidence of cardiovascular diseases is significantly higher in colder seasons compared to warmer seasons, and the mortality rate among cardiovascular disease patients is also significantly elevated during colder seasons (27, 28). This suggests that lower environmental temperatures may be one of the important contributing factors to adverse cardiovascular events (12, 13, 26, 29). Indeed, a small-sample clinical study conducted in Germany found that the incidence of AAD increases by approximately 10% during winter compared to summer (30). Our study had also confirmed the winter season and cold temperature were strongly associated with the increased incidence of AAD onset, and these data were similar with some existing researches (10, 31). The most comprehensive evidence to date comes from Zhang et al. (31), who analyzed 40,270 AAD cases across 313 Chinese cities and reported a nearly linear increase in AAD risk with decreasing temperature, attributing approximately 23.13% of AAD cases to low temperature and 1.58% to temperature decline. While that study provided robust nationwide estimates, its broad geographic scope may have diluted region-specific risk patterns, particularly in areas with extreme cold climates. Our study, though smaller in sample size (*n* = 1,384), offers a decade-long perspective from a severe cold region, capturing climatic exposures seldom represented in national registries. Notably, our cohort from Harbin—a city with winter temperatures routinely below −20 °C and a half-year centralized heating system—provides a unique lens through which to examine the cardiovascular impact of sustained extreme cold and rapid thermal transitions (17).

In terms of the magnitude and lag pattern of extreme cold risk, our study found that extreme cold (−23.4 °C) produced a significant risk on the exposure day itself (lag 0, RR = 2.25), with rapid attenuation thereafter. This acute and transient pattern resembles the findings of Yu et al. (11), who reported that the risk from extremely cold temperature (0.5 °C) was concentrated on the day of exposure (lag 0) and decayed within 2 days. However, the risk magnitude observed here is higher, possibly due to the more extreme cold exposure (−23.4 °C), which may elicit a more intense acute physiological response. Compared with some studies on myocardial infarction that reported cold effects lagging for several days or even weeks (10, 23), the lag effect of low temperature alone in our study was relatively shorter (about 3 days). This may reflect the fact that AAD, as an emergency triggered instantaneously by an intimal tear, depends more on immediate hemodynamic upheaval (1), whereas myocardial infarction involves processes such as plaque destabilization and thrombus formation (32) that require longer accumulation time. Besides, regarding the MMT, previous studies in temperate or subtropical regions have reported MMTs mostly between 24 °C and 28 °C (9–11). Our study identified an MMT of 25.9 °C for the Harbin region. While this value falls within the previously reported range, its contextual meaning is distinctly different. In regions with warm winters, 25.9 °C typically occurs during spring or autumn and is considered a comfortable temperature. In Harbin, however, days with a mean temperature reaching or exceeding 25.9 °C are exceedingly rare and occur mainly during the brief peak of summer (33). This indicates that for Harbin residents, the environmental temperature for most of the year lies below their theoretical optimum, implying chronic exposure to a low-temperature background that may elevate AAD risk. This finding suggests that physiological adaptation and risk perception related to temperature exhibit significant regional specificity (34, 35). Although residents of cold regions display behavioral adaptations to severe cold (36), such as heavy clothing and indoor heating, their cardiovascular systems may lack complete physiological mechanisms to counteract cold stress, rendering even relatively mild low temperatures potentially harmful.

A key finding of this study is the substantial risk posed by sharp temperature drops and their unusually prolonged lag effect. Notably, negative temperature change appears particularly hazardous, with a single-day drop of 9.2 °C associated with an approximately 9.57-fold increase in AAD risk; this elevated risk persisted for up to 15 days, indicating a sustained pathophysiological disturbance. While prior studies have reported an association between temperature change and AAD (9–11, 31), ours not only confirms this link in a severely cold region but also reveals a significantly greater risk magnitude and duration. The nearly tenfold increase in risk from a 9.2 °C drop and its 15-day effect pattern are novel observations. Several mechanisms may explain these findings. First, a sharp drop from an already very low baseline likely presents a more severe physiological challenge than an equivalent drop from a milder temperature, as it combines the stresses of both extreme cold and rapid change. Second, chronic exposure to severe cold may impair endothelial function and reduce vascular elasticity, thereby limiting the capacity to buffer sudden temperature fluctuations. This impairment increases the risk of pathological vasoconstriction and blood-pressure volatility following a sudden drop. Third, the prolonged lag effect suggests that a single sharp temperature drop may initiate a sustained pathophysiological disturbance, such as persistent inflammation, oxidative stress, autonomic dysfunction, or a hypercoagulable state. These alterations could persist beyond temperature normalization, leaving the aortic wall vulnerable.

Furthermore, a distinctive regional pattern emerged, with the number of AAD cases during the prolonged centralized heating period (approximately 6 months) significantly exceeding that of the non-heating period (31). Although heating alleviates indoor cold exposure, it generates steep indoor-outdoor temperature gradients, frequently surpassing 40 °C, which may exacerbate cardiovascular strain through repeated vasoconstriction and dilation cycles (37). This repetitive thermal stress can induce abnormal fluctuations in endothelial shear stress, thereby accelerating endothelial fatigue and dysfunction (38). Consequently, the high incidence during the heating period may not simply reflect low winter temperatures but could stem from combined exposure to pronounced indoor-outdoor temperature differences. This finding implies that public-health interventions in severely cold regions should incorporate, in addition to cold protection, health education on managing environmental transitions smoothly and maintaining adequate indoor humidity.

The triggering of AAD by cold exposure is a complex, multi-system process. Based on our results and prior work, we propose a mechanistic framework that spans acute stress and chronic adaptation. The most immediate pathway is an acute-phase response marked by sympathetic storm (39) and hemodynamic upheaval (40). Cutaneous cold receptors signal the hypothalamic thermoregulatory center, provoking intense sympathetic excitation that elevates heart rate, augments myocardial contractility, raises cardiac output, and induces widespread vasoconstriction, thereby sharply increasing peripheral vascular resistance (41) and central arterial pressure (42). Synergistic activation of the renin-angiotensin-aldosterone system further elevates blood pressure (43). In individuals with pre-existing media degeneration, atherosclerotic plaques, or congenital structural weakness such as Marfan syndrome, this abrupt rise in arterial wall stress can directly cause intimal tearing and initiate dissection. The pronounced risk on the day of exposure in our analysis strongly supports the primacy of this acute hemodynamic mechanism.

Beyond these immediate effects, cold acts as a biological stressor that can induce systemic low-grade inflammation and oxidative stress (44). Exposure elevates circulating levels of pro-inflammatory cytokines, including IL-6, TNF-α, and CRP, while also boosting ROS production via pathways like NADPH oxidase activation and potentially impairing antioxidant defenses (45). Within the aorta, inflammation and oxidative stress promote dissection by directly injuring endothelial cells (46), and degrade media elastic fibers and collagen, and inducing phenotypic switching, apoptosis, or senescence of VSMC (47). The protracted risk period, lasting up to 15 days in our study and particularly evident for type A dissection, may relate closely to a state of low-grade inflammatory and oxidative damage initiated by a single intense cold stimulus. This persistent biological milieu can maintain the aortic wall in a vulnerable state, extending the risk window well beyond the normalization of blood pressure.

## 6 Limitation

Several limitations must be acknowledged. First, as a single-center retrospective study, our cohort may not be fully representative of all populations in cold temperate regions, and we cannot exclude unmeasured confounding factors such as individual activity patterns, indoor temperature, and air-pollution exposure. Second, exposure assessment relied on fixed-site meteorological stations rather than personal monitors, potentially introducing non-differential misclassification and biasing estimates toward the null. Third, limited subgroup sample sizes prevented exploration of effect modification by aortic dissection subtype (Stanford A vs. B) or by genetic background. Future prospective, multi-center studies that incorporate biomonitoring of inflammatory and oxidative markers alongside individual-level exposure data are needed to validate these associations and elucidate the underlying biological pathways. Fourth, our definition of the heating period used fixed calendar months as a proxy for the centralized heating season; while this serves as a reasonable indicator, it cannot precisely quantify the actual daily indoor-outdoor temperature gradient experienced by each individual. Finally, we acknowledge that some point estimates for extreme temperature changes are accompanied by notably wide confidence intervals. This reflects the very small number of AAD events occurring on days with such extreme temperature drops in our single-center dataset. While the continuous exposure-response curves derived from the DLNM provide more stable and reliable risk estimates across the full exposure range, the imprecision at extreme tails warrants cautious interpretation of those specific point estimates. Future large-scale multicenter studies, ideally with prospective designs and larger sample sizes, are needed to validate these findings and to yield more precise estimates at the extremes of temperature variability.

## 7 Conclusion

In conclusion, this study provides novel evidence that in a cold temperate region, both low ambient temperature and sudden temperature drops are significant environmental triggers for AAD, with effects that are more acute and prolonged than previously recognized in milder climates. These findings underscore the importance of developing climate-tailored prevention strategies and enhancing clinical vigilance in populations exposed to severe and fluctuating cold.

## Data Availability

The original contributions presented in the study are included in the article/Supplementary material, further inquiries can be directed to the corresponding authors.
