# Supplementary material for: Low ambient temperature and sudden temperature drop increases the incidence of acute aortic dissection: a retrospective analysis from the northeast region of China
Source: Front Public Health. 2026 May 29;14:1848286. doi: 10.3389/fpubh.2026.1848286 (PMC13260531; doi:10.3389/fpubh.2026.1848286)
Supplement: Supplementary file 1 [file Data_Sheet_1.pdf]

Supplementary Materials

Title: Low Ambient Temperature and Sudden Temperature Drop Increases the Incidence of Acute Aortic Dissection: A retrospective analysis from the northeast region of China

Runing Title: Low Ambient Temperature Increases the onset of Acute Aortic Dissection

Zhenyu Liao, MS*[1]; Zhaorui Liu, MS*[1]; Yue Ding, MS*[1]; Ying Liu, MS[2]; Tao Song, MD, PhD[1], Yike Wang, MS[1]; Ning Zhang, MS[1]; Chao Liu, MS[1]; Zhaoxin Fan, MS[3]; Xin Zhang, MS[4]; Haiyu Zhang, MD, PhD[#1,5]; Song Zhang, MD, PhD[#1,6,7]

[1]Department of Cardiology, the First Affiliated Hospital, Harbin Medical University, Harbin 150001, China; [2]Department of Anesthesiology, Heilongjiang Provincial Hospital, Harbin 150036, Heilongjiang, China; [3]Department of Neurosurgery, the First Affiliated Hospital, Harbin Medical University, Harbin 150001, China; [4]Heilongjiang Provincial Center for Disease Control and Prevention, Harbin, 150001, China; [5]Key Laboratory of Cardiovascular Disease Acousto-Optic Electromagnetic Diagnosis and Treatment in Heilongjiang Province, the First Affiliated Hospital of Harbin Medical University, Harbin, 150001, China; [6]NHC Key Laboratory of Cell Transplantation, The First Affiliated hospital of Harbin Medical University, Harbin, Heilongjiang 150001, China; [7]Key Laboratory of Cardiac Diseases and Heart Failure, Harbin Medical University, Harbin 150001, China.

*These authors contributed equally to this work.

[#]Corresponding author:

Department of Cardiology, The First Affiliated Hospital, Harbin Medical University, Youzheng Street 23#, Nangang District, Harbin 150001, China.

E-mail addresses: 123zhangsong321@163.com (Song Zhang) and zhanghaiyu819@163.com (Haiyu Zhang)

Supplemental Figure and Figure legends

Supplemental Figure-1.

Supplemental Figure-1

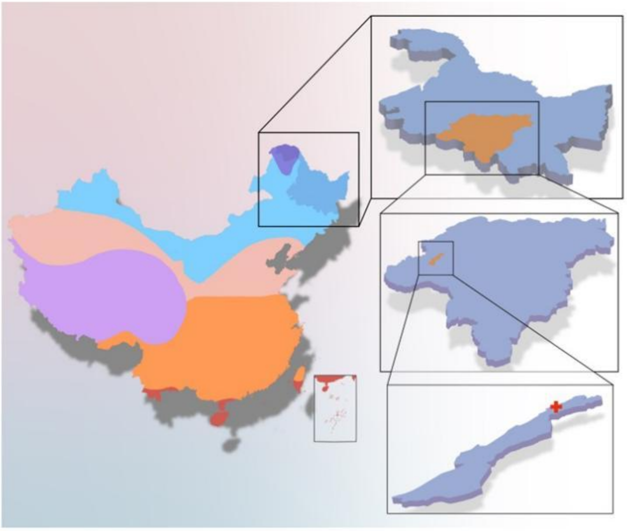

The left panel presents a simplified map of China, where different colors represent the climatic zones of various regions: red for tropical, orange for subtropical, purple for plateau climate zone, pink for warm temperate, light blue for moderate temperate, and dark blue for cold temperate. The geographical administrative boundary of Heilongjiang Province is outlined in blue. The right panel sequentially illustrates the geographical locations of Heilongjiang Province, Harbin City, and the medical center. The urban area of Harbin is highlighted in yellow, and a red cross indicates the geographical location of the medical center within Harbin City.

Supplemental Figure-2. The temperature data corresponding to all observational dates included in the analysis.

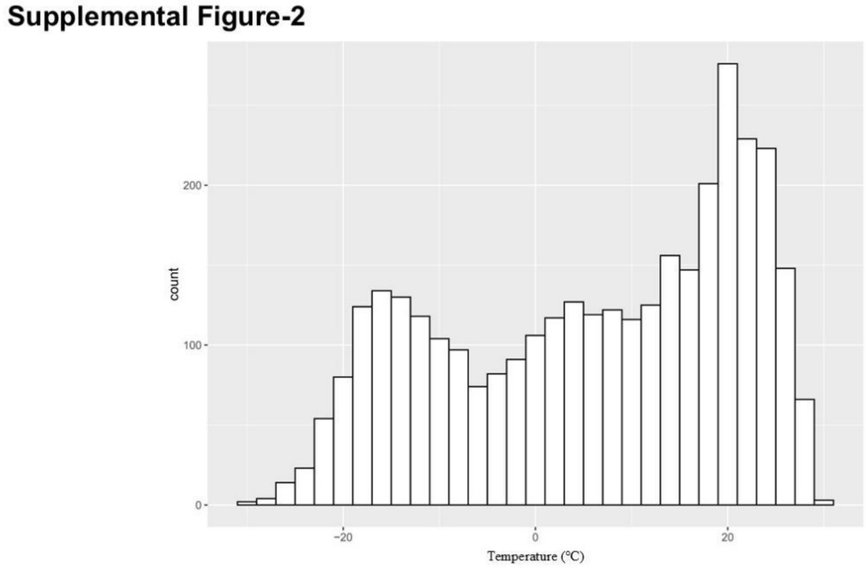

Supplemental Figure-3. AAD cases in Harbin, China from 2014 to2023.

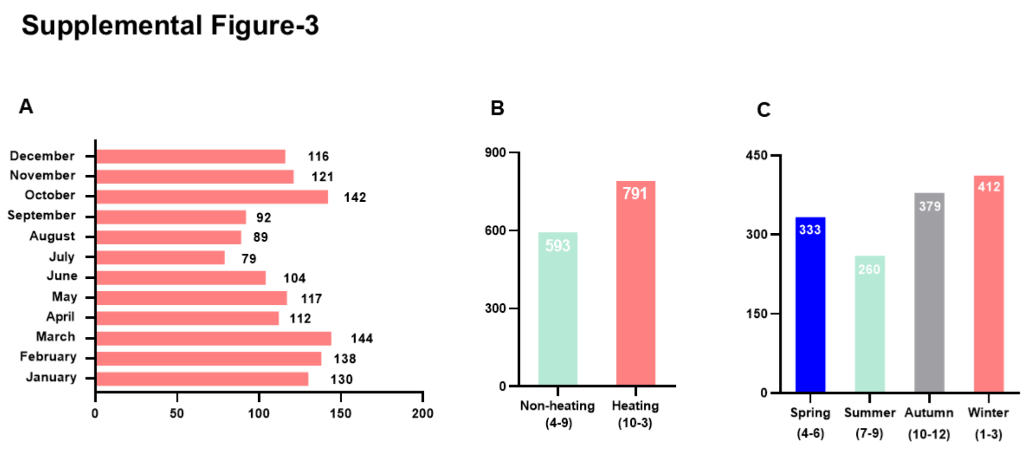

(A) Monthly AAD cases in the medical center region from 2014 to 2023**; (B);** The AAD cases of Heating and Non-Heating period in Harbin from 2014 to 2023; **(C)** The seasonal distributions of AAD cases in Harbin from 2014 to 2023.

Supplemental Figure-4. Bi-dimensional exposure-lag-response relationships between onset of AAD and daily mean temperature displayed in contour plots and 3-D graphs.

Supplemental Figure-4

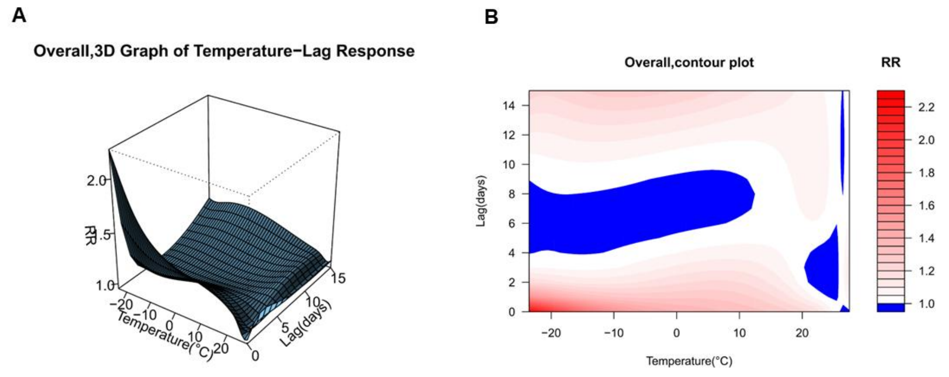

The 3-D graphs and contour plot could not show 95% confidence intervals of the fitted results.

Supplemental Figure-5.

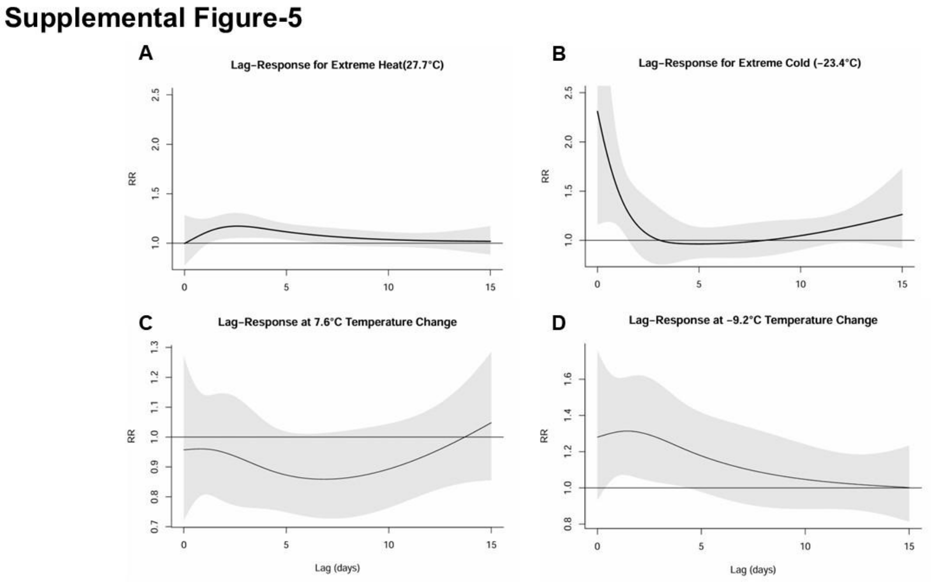

Lag–response curves showing the RRs and 95% CI of AAD onset at extreme temperature and TCN exposures, adjusted for ozone and fine particulate matter. **(A)** Extreme heat: RR at 27.7°C (99th percentile) vs. referent 25.9°C. **(B)** Extreme cold: RR at −23.4°C (1st percentile) vs. referent 25.9°C. **(C)** Extreme positive TCN: RR at 7.6°C (99th percentile) vs. referent 0°C. **(D)** Extreme negative TCN: RR at −9.2°C (1st percentile) vs. referent 0°C. In all panels, the black solid line represents the mean risk estimate, and the gray shading represents its 95% CI. AAD: acute aortic dissection. CI: confidence interval. RR: relative risk. TCN: temperature change between neighboring days.

Supplemental Tables and Table legends

Supplemental table-1. The overall lag-cumulative exposure-response relationship between daily mean temperature and the occurrence of AAD.

Temperature(℃)	RR	RR (95%CI)
-23	7.67	7.67 (2.32-25.37)
-22	7.42	7.42 (2.26-24.38)
-21	7.17	7.17 (2.20-23.30)
-20	6.91	6.91 (2.16-22.13)
-19	6.67	6.67 (2.12-20.94)
-18	6.44	6.44 (2.09-19.83)
-17	6.22	6.22 (2.05-18.86)
-16	6.02	6.02 (2.01-18.04)
-15	5.85	5.85 (1.97-17.37)
-14	5.70	5.70 (1.93-16.81)
-13	5.56	5.56 (1.89-16.32)
-12	5.43	5.43 (1.86-15.89)
-11	5.32	5.32 (1.83-15.50)
-10	5.23	5.23 (1.80-15.14)
-9	5.14	5.14 (1.78-14.80)
-8	5.06	5.06 (1.77-14.48)
-7	4.99	4.99 (1.75-14.18)
-6	4.92	4.92 (1.74-13.88)
-5	4.86	4.86 (1.74-13.60)
-4	4.81	4.81 (1.73-13.32)
-3	4.75	4.75 (1.73-13.06)
-2	4.70	4.70 (1.73-12.80)
-1	4.66	4.66 (1.73-12.56)
0	4.61	4.61 (1.73-12.32)

1	4.56	4.56 (1.72-12.09)
2	4.52	4.52 (1.72-11.86)
3	4.47	4.47 (1.72-11.64)
4	4.42	4.42 (1.71-11.42)
5	4.37	4.37 (1.70-11.19)
6	4.31	4.31 (1.70-10.96)
7	4.25	4.25 (1.69-10.72)
8	4.19	4.19 (1.67-10.48)
9	4.12	4.12 (1.66-10.21)
10	4.04	4.04 (1.64-9.94)
11	3.96	3.96 (1.63-9.65)
12	3.88	3.88 (1.61-9.35)
13	3.79	3.79 (1.59-9.04)
14	3.69	3.69 (1.56-8.71)
15	3.58	3.58 (1.53-8.39)
16	3.47	3.47 (1.50-8.07)
17	3.36	3.36 (1.45-7.76)
18	3.24	3.24 (1.40-7.48)
19	3.11	3.11 (1.34-7.23)
20	2.98	2.98 (1.26-7.03)
21	2.82	2.82 (1.17-6.75)
22	2.55	2.55 (1.08-5.99)
23	2.13	2.13 (1.00-4.54)
24	1.60	1.60 (0.93-2.74)
25	1.14	1.14 (0.90-1.43)
26	1.01	1.01 (0.97-1.04)
27	1.48	1.48 (0.99-2.21)
28	4.86	4.86 (1.36-17.34)

RRs for lower and higher temperature were estimated relative to the reference temperature of 25.9 °C. In this table, RRs are based on 15 days of temperature exposure. RR, Relative risks.

Supplemental table-2. The overall lag-cumulative exposure-response relationship between extremely temperature and the occurrence of AAD.

Lag	RR of the heat effect	95% CI	RR of the cold effect	95% CI
0	0.988	(0.810, 1.204)	2.252	(1.205, 4.208)
1	1.087	(0.989, 1.195)	1.545	(1.210, 1.974)
2	1.143	(1.047, 1.247)	1.194	(0.907, 1.573)
3	1.146	(1.053, 1.248)	1.049	(0.790, 1.394)
4	1.121	(1.048, 1.200)	0.998	(0.807, 1.234)
5	1.093	(1.032, 1.158)	0.979	(0.833, 1.152)
6	1.071	(1.010, 1.135)	0.972	(0.827, 1.142)
7	1.053	(0.991, 1.120)	0.974	(0.819, 1.157)
8	1.04	(0.978, 1.107)	0.984	(0.825, 1.173)
9	1.031	(0.972, 1.094)	1.002	(0.848, 1.183)
10	1.025	(0.970, 1.084)	1.026	(0.885, 1.190)
11	1.022	(0.969, 1.078)	1.057	(0.926, 1.207)
12	1.021	(0.963, 1.081)	1.094	(0.952, 1.256)
13	1.021	(0.952, 1.095)	1.135	(0.950, 1.356)

14	1.022	(0.935, 1.117)	1.181	(0.929, 1.501)
15	1.024	(0.916, 1.144)	1.229	(0.897, 1.684)

The effects of cold and heat on AAD occurrence were calculated by comparing the risks at the 1st (-23.4 °C) and 99th (27.7 °C) percentile temperatures to the reference temperature. RR, Relative risks.

Supplemental table-3. Relative risks and 95% confidence intervals of AAD associated with each TCN.

TCN	RR	RR (95%CI)
-9	9.566	(1.208, 75.762)
-8	6.375	(1.117, 36.389)
-7	4.374	(1.020, 18.767)
-6	3.103	(0.923, 10.436)
-5	2.285	(0.835,6.252)
-4	1.755	(0.768, 4.008)
-3	1.411	(0.734, 2.714)
-2	1.193	(0.745, 1.910)
-1	1.065	(0.825, 1.376)
0	1.000	(1.000, 1.000)
1	0.947	(0.730, 1.228)
2	0.862	(0.533, 1.395)
3	0.743	(0.381, 1.446)
4	0.608	(0.261, 1.417)
5	0.475	(0.167, 1.356)
6	0.356	(0.097, 1.303)

The risks were presented as RR and 95% CIs of acute aortic dissection at each TCN along the 1st and 99th percentile of TCN distribution compared to the referent TCN (0°C) cumulated over lag 0-15 day.

Supplemental table-4. Stratified analysis of cumulative risk of AAD at extreme cold and heat.

Subgroup	N	Extreme cold<br>RR (95%CI)	Extreme heat<br>RR (95%CI)
Overall	1384	3.50 (1.56,7.85)	2.05 (1.24,3.42)
Age			
<60	904	3.55 (1.32,9.56)	2.57 (1.29,5.13)
>=60	480	2.64 (0.75,11.49)	1.81 (0.77,4.25)
Sex			
male	974	2.76 (1.04,7.31)	1.88 (0.94,3.74)
female	410	7.52 (1.68,33.64)	2.34 (1.00,5.46)

RR, risk ratio; 95% CI, 95% confidence interval.

Supplemental table-5. Stratified analysis of cumulative risk of AAD at extreme negative TCN.

		TCN (-9.2℃)
Subgroup	N	RR (95%CI)
Overall	1384	4.49 (1.71,12.85)
Age		
<60	904	3.53 (0.97,12.82)
>=60	480	8.31 (1.59,43.34)
Sex		
male	974	4.16 (1.24,14.05)
female	410	5.99 (0.97,37.12)

RR, risk ratio; 95% CI, 95% confidence interval; TCN, temperature change between neighboring days.
